# The use of video essays and podcasts to enhance creativity and critical thinking in engineering

**DOI:** 10.1007/s12008-022-00952-8

**Published:** 2022-07-11

**Authors:** Patricia Caratozzolo, Vianney Lara-Prieto, Samira Hosseini, Jorge Membrillo-Hernández

**Affiliations:** 1 Institute for the Future of Education, Tecnologico de Monterrey, Monterrey, Mexico; 2 School of Engineering and Sciences, Tecnologico de Monterrey, Santa Fe Campus. Av. Carlos Lazo 100, Mexico City, Mexico; 3 Writing Lab, Institute for the Future of Education, Tecnologico de Monterrey, Ave. Eugenio Garza Sada 2501, Monterrey, NL Mexico; 4 School of Engineering and Sciences, Tecnologico de Monterrey, Monterrey. Campus Av. Eugenio Garza Sada 2501, Monterrey, NL Mexico; 5 School of Engineering and Sciences, Tecnologico de Monterrey, Mexico City Campus. Av. Del Puente 222, Mexico City, Mexico

**Keywords:** Creative thinking, Twenty-first century skills, Critical thinking in engineering, Higher education, Educational innovation, STEM education

## Abstract

The current demands of the labor market demand a new compendium of skills from engineering graduates. To develop skills at a more complex level, this study analyzed the use of second-generation Video Essays and Podcasts to improve soft skills. The characteristics of students belonging to Generation Z as digital natives were considered and digital platforms were adapted for interaction in social networks to enhance critical thinking and creativity (Criticality). Active learning experiences in different engineering programs were analyzed using the 4-group Solomon methodology with a quantitative design and different assessment instruments were used for Pre-Tests and Post-Tests, including various fluency and originality tests, as well as answering articulation ability tests. and modified VALUE rubrics from the Association of American Colleges and Universities (AAC&U). Our results clearly suggest that the use of these two tools substantially improves understanding of scientific concepts in engineering subjects, a greater ability to develop increasingly in-demand skill sets, and a greater awareness of creative thinking competence.

## Introduction

The study of the development of soft skills in engineering, especially critical thinking and creative thinking, has proven to be of special interest, first in relation to the Fourth Industrial Revolution and then, due to the outbreak of the COVID-19 crisis [1, 2]. For almost two years, from March 2020 to the end of 2021, most Higher Education Institutions (HEIs) around the world struggled not only with teaching classes remotely but also with the incorporation of students belonging to the Generation Z, which strengthened the need for innovative approaches so that these skills are developed effectively and lastingly, considering the particular characteristics of Generation Z youth [3, 4]. As part of the Education 4.0 theoretical framework, both employers and international accreditation agencies have focused on the development of these competencies and propose that they be formally included in engineering programs. Additionally, educational innovation strategies suggest including new cognitive and metacognitive tools to achieve the full development of soft skills in Generation Z engineers. Among the triggering factors of these proposals are: first, the strong consolidation of the communication in social networks, where private and public affairs converge indistinctly; and secondly, the exponential development of technological platforms, which mean a big difference in the way that Generation Z engineering students develop their cognitive skills, compared to the previous generation, the Millennials [3, 4].

The development of resources -in social networks and information technologies- that was unleashed as an avalanche due to the COVID-19 pandemic in all areas of society, was incorporated in an uncontrolled way into HEI's educational environments, with the risk that it would not be possible to guarantee the effective intellectual engagement of students in their own learning process [5]. To correct these harmful effects, all kinds of classroom implementations emerged with the aim of increasing the motivation and commitment of university students, mainly in the first years, when the highest dropout rate is observed [6]. Some of the most interesting implementations were the gamification of courses, the incorporation of educational apps and videos, the use of mobile devices for communication, and the use of augmented reality and virtual reality programs. This phenomenon was especially noticeable in engineering courses due to the use of essential technological platforms in Science Technology Engineering and Mathematics (STEM) programs. This section does not attempt to summarize the entire scope of the literature, but rather to identify key themes related to creativity and critical thinking (criticality) in engineering and provide a narrative description of the gaps of the last four years (2019–2022) with the aim of understanding the design of the new learning approaches proposed in this study (use of Video and podcast):

2019. Chaikovska et al. (2019) recommended university teachers to integrate profession-based podcasts with students of technical universities to improve student communication skills, to provide participants with new vocabulary and to encourage the interaction with engineering community [7]. On the other hand, Hussein et al. (2019) analyzed the public perception and understanding of technological advances in engineering and finds that they are difficult to maintain. The study determined that the rate of dissemination of information is a limiting factor since public policies and practices handle outdated information with respect to cutting-edge technology. This study proposed that the STEM community participate in scientific education and communication practices appropriate for the new digital age through a podcast channel where findings on impact and best practices are shared [8]. Likewise, Sgambi et al. (2019) reported an active didactic experience -from its conception to its development in the classroom with the students- with three main steps: stimulation, practice, and discussion. The study highlighted the relevance of an active didactic experience to improve courses that are generally passively structured and argues that, in an era where the importance of cutting-edge technology is growing, this type of experience, with a low-tech approach, results very suitable to develop the creativity and criticality of the students [9]. Finally, the findings of a study by Miller et al*.* (2019) had important implications for engaging students in interdisciplinary applied research, including fostering critical and creative thinking skills and harnessing technology for the greater good social science by collaborating on the design of mobile device applications that improve the lived experiences of community [10].

2020. The work of Nissenson et al. (2020) detailed the creation and distribution of an audio podcast related to the engineering student experience with the purpose of helping current and future engineering students thrive in college and in their future careers through conversations and interviews with practicing engineers, engineering professors and engineering students [11]. In the same line, Becerra & Almendra (2020) measure the motivation levels of students in the statistical inference module of an engineering course. The data showed a high motivation with the use of podcasts as a means of instruction in the course [12]. Moreover, the goal of the Chiodo et al*.* (2020) was to make students reflect on the role of imagination in various contexts, including scientists and citizens, and to teach them how to strengthen creativity in order to improve the graduate competencies of an engineer [13]. Finally, the study by Wu et al. (2020) introduced project-based learning and SCAMPER teaching strategies in an engineering project course. This project involved students in experimental activities for two semesters, which allowed to analyze the differences between high and low creativity students in terms of cognition, personal motivation and personality traits [14].

2021. Torres-Gómez et al. (2021) demonstrated that improvements in teaching effectiveness are possible through a multi-sensory approach supported by the liberal arts. The methodological design included the development of artistic and literacy skills along with the fundamentals of specific technical topics, which were taught from a qualitative perspective. The project confirmed that this approach increased both the understanding and the evaluation of abstract concepts by stimulating the creativity and curiosity of the students [15]. Qamar et al. (2021) described a strategy to instill critical thinking skills in engineering graduates. Various critical thinking models were explored, and Paul and Elder's model was found to be more suitable for engineering as it provides a good foundation for the way engineers think and focuses on issues such as creativity, design development and professional and ethical issues. During this study, the instructional strategy (especially discussions and interactive sessions) was modified to include aspects of critical thinking [16]. In the same line, Zúñiga-Robles and Truyol (2021) aimed to investigate the benefits of using an innovation "Flipped Classroom + Podcast" in a geophysics course for engineering students. A descriptive research approach was used to determine if the use of podcasts had a positive impact on students' attitudes and if they were perceived as a useful tool in the construction of knowledge, discovering that students did indeed obtain greater motivation and engagement [17]. Aulia and Utami's (2021) assessed the students' *e-*learning knowledge to support the development of twenty-first century learning skills. Several indicators in the development of learning management were studied, including critical thinking, creativity, and communication skills. The results indicated that students' literacy skills can be improved to meet the learning requirements of the twenty-first century [16].

2022. Sudakova's (2022) analyzes the role of creativity in the formation of meta-competences of students and future engineers. She compares examples of successful pedagogical practices that use various forms of interaction between teachers and students in the learning process: discussion, business role-playing games, and video essay [18]. On the other hand, Schiele et al. (2022) proposes minor but important modifications in the design of traditional "World Café" experiences to be fully applicable as a method of academic data collection. Their results include a strong discussion of rigor and relevance in management research and therefore introduce the "research world café" as an academically rigorous data collection method [19]. In addition, Priyadharshini and Parveen (2022) offers the results of the application of a survey on the perceptions and the impact of the podcast on teaching–learning practices. The study focuses especially on the teaching–learning process and analyzes findings that suggest that the contents of podcasts are referred by students especially for the preparation and review of evaluations [20]. Very recently, Zainal et al*.* (2022) analyzes the current requirements to train competent graduates in oral and written communication, who stand out for their twenty-first century skills such as creativity, media and technological literacy. The study proposes that these competencies be comprehensively developed through instructional approaches such as project-based learning. The use of group work video essays in the classroom was examined during this project and was based on an assessment of student video essays and an open interview with students [21].

The review of the studies carried out in the last four years seems to indicate, the use of video and podcast technology is very important for the development of creativity, reliability, and critical thinking of engineering students, which will most likely lead to strengthening the intellectual commitment of Generation Z Engineering students. During this literature review, weaknesses, and gaps in the strategies for developing soft skills in engineering were detected, especially in studies related to the development of critical thinking and its correlations with creative thinking, since some works refer to the infusion of strategies in the classroom and others to the incorporation of more technological tools. The biggest gap detected was trying to develop soft skills through the traditional logical-scientific thinking modality for specific critical thinking skills that include inference, judgment, problem solving and identification of assumptions. The proposal of this study consists of an approach that intellectually engages engineering students and encourages the development of specific creative and critical thinking dispositions, instead of limiting the scope of the approach to separately strengthening creativity and criticality skills.

In the following section, the background related to four aspects of learning in higher education, specifically in engineering, will be presented: (i) the characteristics of Generation Z students; (ii) the dynamics of cognitive processes; (iii) the traditional model of creativity, and finally (iv) the traditional model of criticality.

## Background

### Learning skills of generation Z students

Generation Z (Gen Z) is defined as people born between 1995 and 2010. Gen Z individuals were exposed to many digital technologies and developed different learning characteristics compared to the previous generation, the Millennials. Gen Z students make intensive use of digital technologies not only for formal learning, but also for informal training and for their interaction in social networks [22]. Gen Z students are highly skilled technology users who rely heavily on information from social media and the Internet, making their learning processes deeply dependent on all types of modern technologies: online platforms, social networks, digital chats, online games and -forced by the COVID-19 pandemic- they have received two years of online education, D-Learning.

Gen Z students are characterized by a special typology of "digitalized" consciousness and thought that had not been identified in Millennials, which is called *clip thinking*, which implies that their thought has a segmented consistency. The effectiveness of educational teaching strategies will be directly conditioned to this segmented nature, form and content that form the thought processes of students. In the environment of engineering education there is no unified interpretation of the concept of *clip thinking*, but it is possible to identify its main characteristics and distinctive properties [23]:

- Ability to perceive large flows of digital information, such as electronic, audio or video information
- Habit of consuming large amounts of fragmentary information from social networks without reflecting on whether it is fake or tendentious information
- Immediate perception of fragments of information dispersed in the flow, and subsequent interpretation of them as if it were a coherent speech
- Strong preference for high-speed visual perception of information at a superficial level, without awareness of deep content
- Predominance of concrete thought over abstract thought, with a preference for non-textual information
- Ability to flow naturally from a real environment to a virtual one and vice versa,
- Lack of development of reflective plans based on critical thinking, and weak ability to draw conclusions and consider consequences
- Difficulties in comprehensive analytical perception and logical understanding of phenomena and their relationships
- Minimalism of the lexical corpus and limitations of oral communication

The advancement of technology has contributed to the knowledge and skills gap between Gen Z students and their predecessors, the Millennials. For example, Gen Z individuals are, on the one hand, more pragmatic and aware of opportunities thanks to technology, while on the other hand they are more impatient and have a shorter attention span. The learning characteristics of Gen Z students are modeled according to their personality traits, as they are addicted to technology and speed, independent and individualistic, do not appreciate teamwork, and tend to be dissatisfied [24]. Teaching approaches aimed at Gen Z students must adapt to these personality traits since they have developed a flexible perspective and a preference for non-standard, non-routine and personalized activities that allow them to live innovative and creative experiences.

Gen Z students are immediate feedback oriented and need to access information quickly, so they expect the same real-world circumstances in the classroom. Most of the time, teachers have to deal with the fact that students become impatient if they don't receive immediate feedback and quick information that they are used to during their daily online life. Various studies suggest that the intensive use of the internet and the search for information on the digital network influence the brain activity of Gen Z individuals: the activity is generalized in certain determined areas while those neural pathways, which supported traditional mental functions, weaken and new connections suitable for clip thinking are formed [25]. This new "way of thinking" forces the brain to do intensive work that can impede deep thinking and learning. By prioritizing segmented thinking, the capacity for mental coordination and decision-making is lost. This circumstance could be reversed by promoting spaces for critical reading of academic texts, oral communication experiences among peers, and debates on technical issues in the classroom [26, 27].

### Cognitive process and creative thinking

**Fig. 1:**
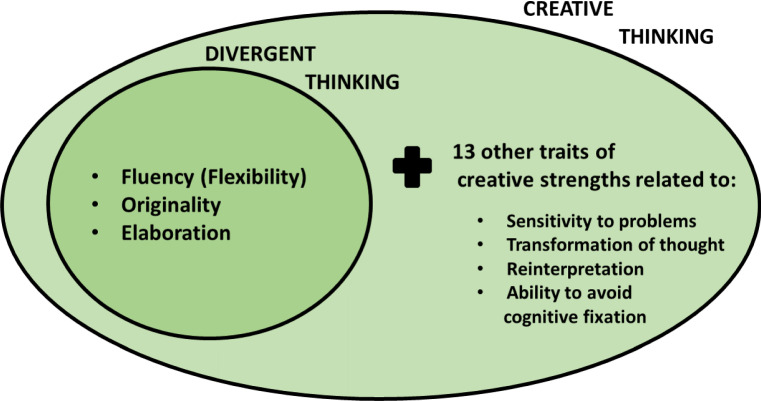
Creative thinking and divergent thinking. as it is shown, creative thinking is a broader concept than divergent thinking

Before choosing methods to evaluate creativity, it is important to be able to understand what the qualities that help students to express their creativity are, that is, those that unravel their cognitive process. It can be said that creative thinking includes divergent thinking, which is formed by three characteristics: Fluency (as the ability to generate many responses or ideas, including flexibility); Originality (as the ability to change form, modify information or shift between thinking modes); and Elaboration (as the ability to express an idea with multiple details). As shown in Fig. 1, creative thinking not only is divergent thinking but also includes other traits of creative strengths, related to problems sensitivity and redefinition of skills such as transformation of thought, reinterpretation and ability to avoid cognitive fixation [28].

**Fig. 2:**
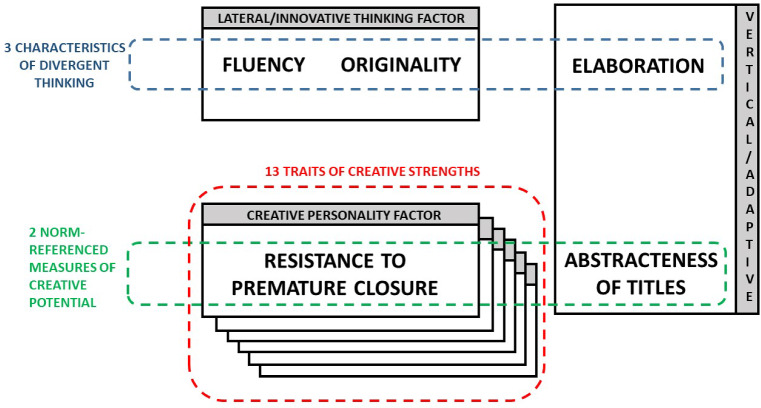
Schematic showing the relationship between different taxonomies for Creativity

Another way to conceptualize Creative Thinking is by considering five subscales: the three characteristics of divergent thinking (*Fluency* + *Originality* + *Elaboration*) and two Norm-referenced measures of creative potential (*Abstractness of titles* + *Resistance to premature closure*). When considering this different taxonomy, two concepts appear that will be very useful to understand how the cognitive process works and how to measure it: lateral and vertical thoughts. Lateral Thinking, also known as Innovative Thinking Factor, is represented by two characteristics: fluency (understood as the amount of relevant ideas) and originality (understood as the amount of statistically uncommon, unusual, unique ideas) [29]. Vertical thinking, also known as adaptive, is represented by two characteristics: *(i)* Elaboration, understood by the number of ideas that arise from others, combining them, adapting them and giving them “another twist” which shows a conviction to be creative; and *(ii)* Abstractness of titles (abstract articulation) which is the ability to carry out the reasoning process that involves synthesis and organization, as well as the ability to distinguish the essence of information and recognize what is important. The abstraction of thought implies "verbal intelligence", with which this characteristic is closely related to critical thinking. A schematic showing these concepts can be seen in Fig. 2.

**Fig. 3:**
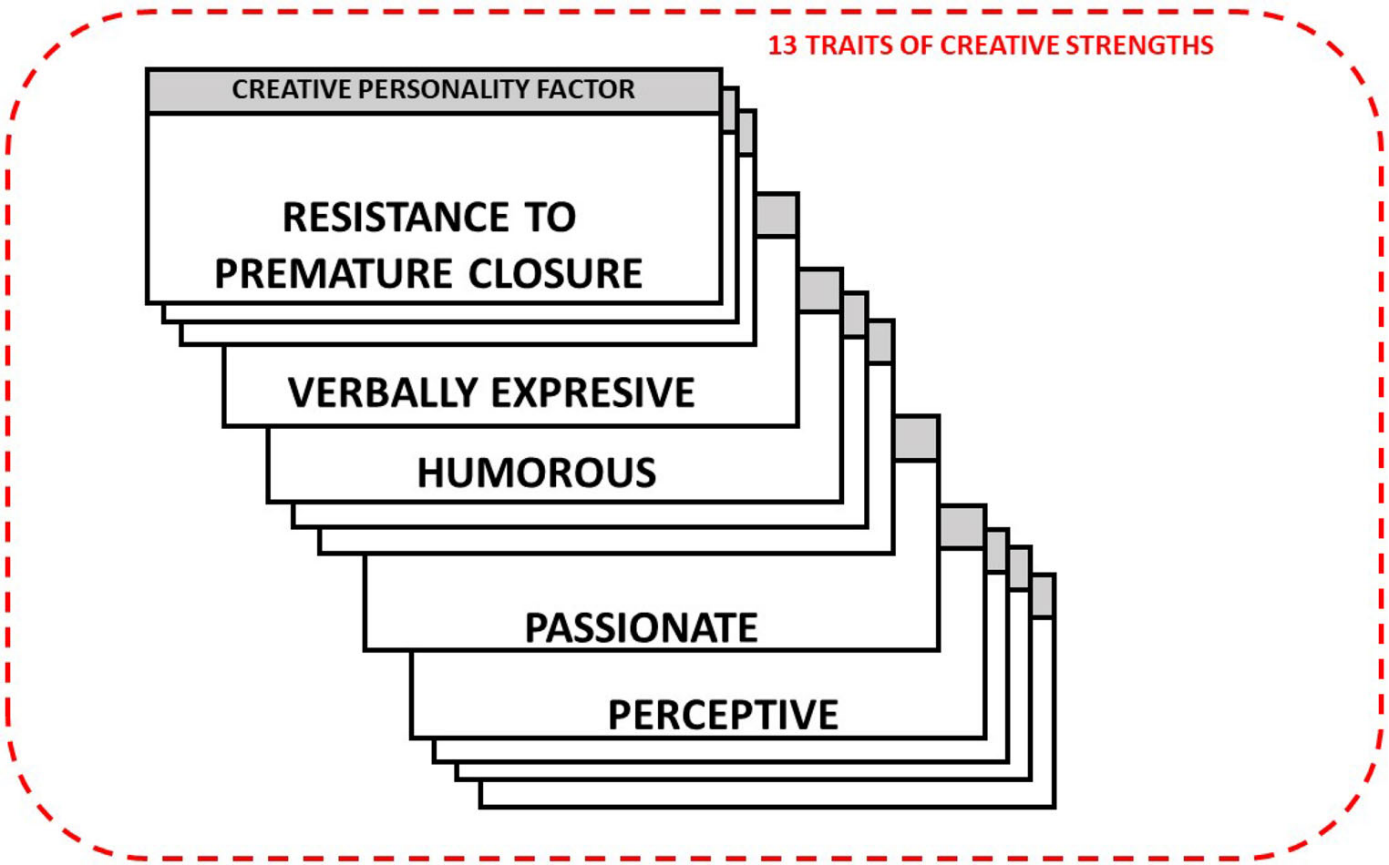
Four traits of creative strengths considered in this study in addition to resistance to premature closure

The factors for a creative personality are represented by: Resistance to premature closure (it is the depth of intellectual curiosity and the level of open-mindedness), and thirteen other Creative Strengths, also known as Personality Traits. There is evidence in Torrance's original studies (1979, 1988 and 1994) that the thirteen subscales are good predictors of Creative Achievement, so it is valid to propose an instructional method for the development of creative thinking through the practice of experiences related to the exercise of only four personality traits shown in Fig. 3: Storytelling Articulateness (verbally expressive), Humorous Ability, Richness of Imagery (passionate) and Colorfulness of Imagery (perceptive) [30].

**Fig. 4:**
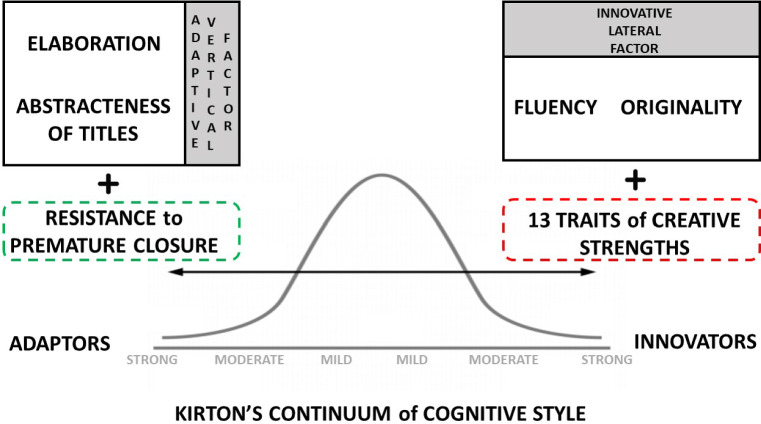
Kirton’ s cognitive style and creativity factors, modified from [32]

In this study it was also taken into consideration that with a two-factor model, more significant results could be obtained than with a single-factor model; Thus, the *Kirton Adaptor-Innovator (KAI)* theory was also considered [31]. According to Kirton, people react to changes with attitudes that define them in two very different positions: Innovators, who prefer to create change by breaking paradigms; and the Adaptors, who prefer to create changes working within the existing paradigm. These two positions can be understood as two opposite ends; however, there is a whole continuum of approaches to creative problem solving known as *Kirton’s continuum* If the composition of any engineering group is analyzed, it is verified that students present differences in cognitive style according to normally distributed continuum, which ranges from strong adaptation on one end to strong innovation on the other, as is shown in Fig. 4. This distribution refers only to the creative style and is independent of the level of creativity, therefore when measuring the correlation between the subclasses of creativity and the level of creative thinking, Kirton’ s theory must be considered.

Adaptors and innovators have different perception of problems and therefore different approaches to solution, with no appreciable differences in the level of creativity of students. Making a parallel between the *KAI* and the divergent-convergent process of *Design Thinking Method*, the innovators will stand out in the divergent stage and the adapters in the convergent. This highlights the usefulness of forming creative teams in a balanced way, taking advantage of the natural composition of groups with participants with cognitive styles from all states. All the elements of a creative team can and should be involved in the divergent-convergent processes, to be able to contribute their points of view, considering that their level of engagement at each stage will depend on factors such as the level of perseverance, motivation and satisfaction with the proposed solutions, and the level of Premature Closure [32]. Although the studies do not reveal differences in the creative potential of Gen Z students due to their *KAI* personality, there are implicit theories of creativity among university professors that lead to a misinterpretation of the evaluation of creative thinking, especially regarding STEM students. Many teachers misunderstand the creative potential of students due to the confusion produced by different personality traits related to creativity, since they relate the level of creativity to the cognitive style [33].

### Traditional creativity model

Creative ideas arise from the synergy of many sources, and not only from the mind of an isolated person. Because students are part of a learning group, social interaction ability should be considered part of the creative process. Therefore, to strengthen the development of this individual skill, each student should be considered to be immersed in a Creative Model in the style proposed by Csikszentmihalyi [34–36]. This model states that a person's creative process cannot be analyzed as if the individual were isolated but should be considered in relation to the environment in which individuals operate, that is, *Person* interacts with *Domain* and *Field*. The *Field* is what we usually call culture, a series of rules or symbolic knowledge shared by a particular society; The *Person* is who exercises a creative process and who wants to create a new *Field* or change it to show their ideas and products; and the *Domain* is which includes those individuals who act as gatekeepers to give access to the *Field*. Its function is to decide whether an idea or product of a *Person* should be included in the *Field*. One of the most recent studies on creative thinking in engineering is that of Santos et al*.* [37] that proposes a methodology to introduce creativity and innovation techniques in the engineering process. The method uses a variety of creative techniques that are considered appropriate for the different stages of the process and draws inspiration from existing creative problem-solving methods and techniques.

### Traditional criticality model

Critical thinking is the active, persistent and careful analysis of any belief or supposed form of knowledge in light of the fundamentals that support it and the conclusions from which it arises [38]. In this study, it was taken into consideration the theory of the relationship between critical thinking and the educational process presented by John Dewey in order to develop students' ability to think reflexively [39]. Currently, it is very difficult to counteract the natural tendency of people to generate incorrect thinking; what Francis Bacon called *idols*, which attract the mind to false paths. This model is made up of four elements: the *Tribe*, that encourages individuals to make mistakes whose roots are in human nature in general, for example with superstitions, fantasies and myths; the *Market*, that generates in individuals the erroneous methods that come from exchange and language, such as misunderstandings and prejudices; the *Cavern*, whose incitements to error have strictly individual causes, such as traumas and frustrations; and the *Theater*, that produces those errors that have their origin in fashion or the general opinion of society, generating trends and political positions [40]. One of the most recent studies on critical thinking in engineering is that of Giuliano et al*.* [41] that develops some ideas to strengthen the inclusion of humanistic knowledge in science education. The study includes a novel formal definition of the term "judgment" to illuminate the conceptual links between technical rationality and critical thinking in the context of the engineering profession.

### Proposed approach: creativity in criticality

Different studies reinforce the idea of Vigotsky and Csikszentmihalyi about the influence of the social interaction on reflective thinking and the development of creative process [42, 43]. Generation Z students have idols -Market, Theater, Tribe and Cavern- which are particular and different from those expressed by previous generations. For the practical application of the approach in the present study, some considerations were made regarding how the two models could be related: First, the *Person* is represented by the *Cavern* (is each engineering student); Secondly, the *Tribe* is represented by the *Domain* (are all the teachers, instructors, classmates, jury contests, publishers of scientific publications, synod and advisors); and finally, both the *Market* (the areas of cutting-edge knowledge such as virtual reality, augmented reality, artificial intelligence, IoT, automated vehicles, etc.) and the *Theater* (current trends such as sustainability, climate change, energy efficiency, social networks, circular economy, transactional energy, etc.) are represented by the *Field*.

**Fig. 5:**
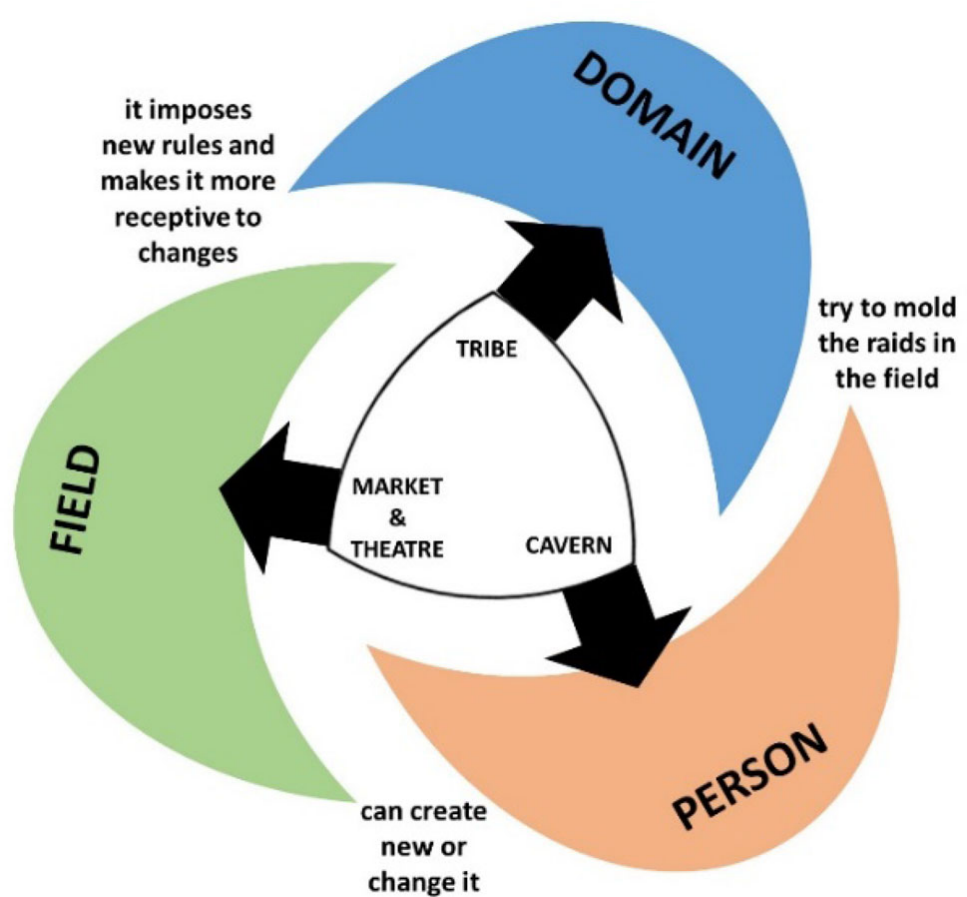
Creativity and false idols represented with a *Reuleaux Triangle* of beliefs

A systematic and theoretical analysis was carried out in this study, considering an extended approach between both systems, creativity and criticality, to find metacognitive tools that will improve the teaching-learning process considering the development of both soft skills in a joint and synergistic way. Figure 5 shows how both model systems can be considered together using the hypothetical relationship mentioned in the previous paragraph. The Idols model can be represented by a *Reuleaux Triangle*, which imposes its beliefs -it pushes out the three circles of Csikszentmihalyi's creativity model- interfering in the free creative flow.

A creative process is verified when a Person, using symbols and languages of the *Market*, and imposing her/himself to the fashions and trends of the *Theater*, manages to place a new idea or product in the Field -overcoming the traumas and frustrations of his own *Cavern*- with the approval of the Domain, which accepts it despite the superstitions and myths of the *Tribe*. Therefore, it is not enough to just unravel the internal mental process of the students, but it is necessary to analyze the process in the context of the classroom and the academy, to understand how creative thinking can be enriched and what possible reluctances are interspersed in their flow path. The innovative approach developed in our study allows to distinguish the *Cognitive Process*, internal to the *Cavern;* the FLOW path, going from the Domain to the Field (across the Person), and finally, the *reluctances* that any idea or new product must overcome to complete the creative process successfully. It can be verified that Idols Model does not represent impermeable barriers but strong reluctances to the free flow of ideas [28, 44, 45].

Jerome Bruner points out that there are two modalities of cognitive functioning of thought, and each of them offers characteristic ways of constructing reality and ordering experience [46, 47]: *The logical-scientific modality* (critical thinking) tries to fulfill the ideal of a mathematical, formal system of description and explanation. It uses the categorization or conceptualization and the operations by which the categories are established, represented, idealized and related to each other in order to build a system. *The artistic-narrative modality* (creative thinking), on the other hand, looks for connections between two events and uses procedures to establish the likelihood, not the truth. The artistic-narrative modality deals with students’ ability to develop new technological products through conversion of the imaginative concepts into a dependable reality. Although shifting may occur in either direction, training for shifting from critical thinking to creative thinking has been unfairly suppressed in engineering programs mainly so as not to lose the rigor of the search for empirical truth. There is a false perception that engineers should use only the principles of criticality instead of creativity, however, emotions and aesthetics enrich the solutions provided to the problems since it is impossible to judge critically what is based solely on rational arguments [48, 49].

Because Gen Z students are part of the learning group, *Shifting Mode* -the ability to shift between thinking modalities- should be considered part of the creative process. Our study considered the *Stiffness* of the Shifting Mode according to the magnitude of two cognitive biases: *(i) Premature closure* (as the cognitive bias that causes the student to not consider reasonable alternatives after an initial diagnosis of a problem is made); *(ii) Cognitive fixation* (as the cognitive bias that causes the student to evaluate the functionality of an object only in the way it is traditionally used). The development of higher levels of students' creative potential seems to require two catalysts to counteract these biases: *(i)* The continued exposure to diversified experiences that help weaken the restrictions (cognitive fixation) imposed by the Domain*;* and *(ii)* the experience of challenging experiences that help strengthen the ability to persevere in the face of obstacles (Premature Closure) that Field represents.

The most innovative element of our approach is the introduction of the concept of metacognition as part of the theoretical framework of the learning process of Gen Z engineering students. Metacognition or *thinking about thinking* is a multidimensional set of general, rather than domain-specific skills. Metacognitive knowledge consists of cognitive learning strategies, which students can use to regulate the process of knowledge acquisition. Intellectual engagement does not occur automatically: successful engagement depends not only on the cognitive effort but also on the metacognitive processing, which in turn depends on the development stage of the student. Due to the fact that in the same classroom students with different levels of cognitive development coexist, we have considered two fundamental concepts derived of Vygotsky's work, *Scaffolding and Zone of Proximal Development* [50]*.*

**Fig. 6:**
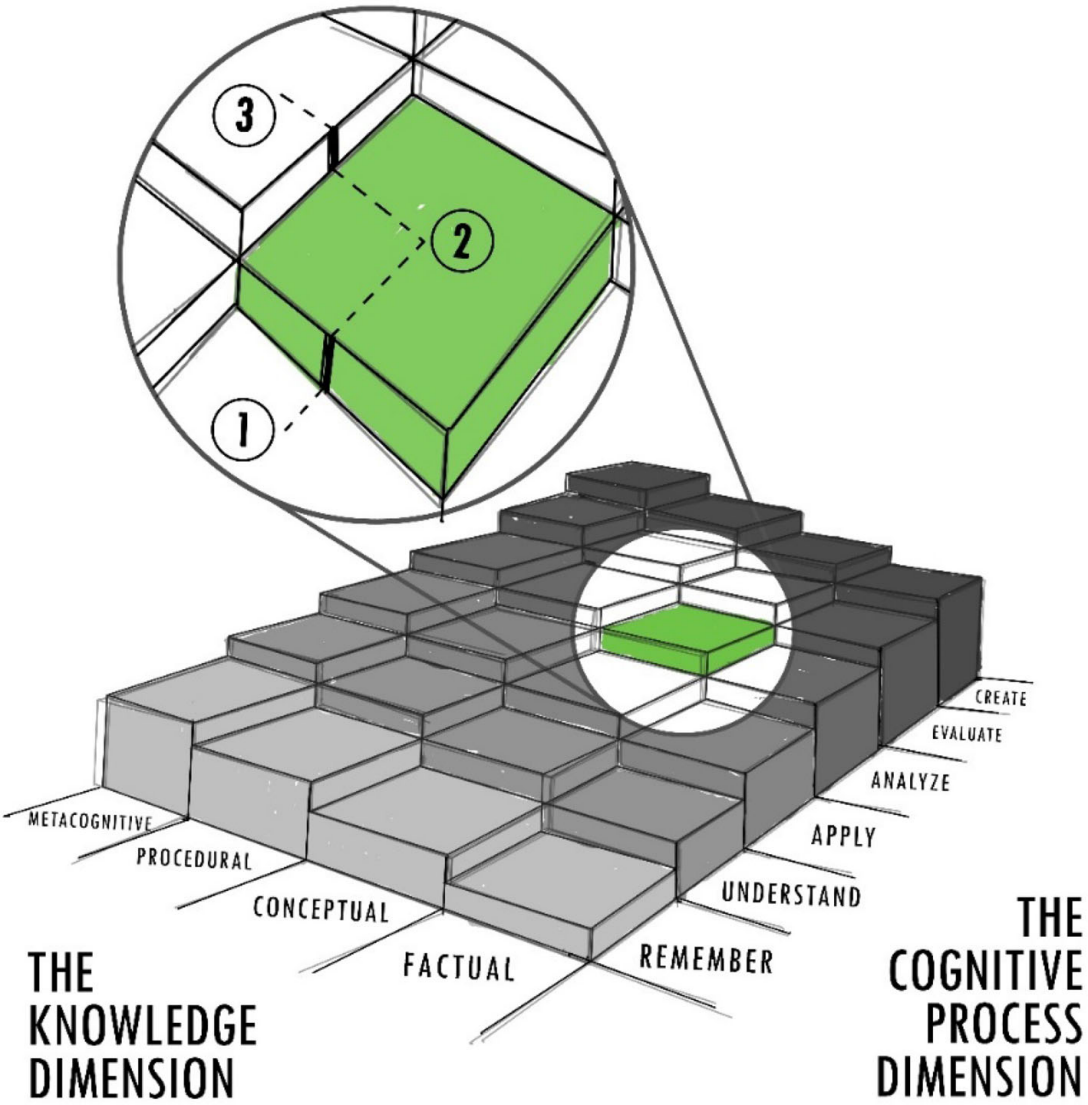
The multi-level tiered yard of the creativity in criticality approach

A graphical way of understanding our novel approach is to imagine it as a three-dimensional space, a kind of multi-level *tiered yard*: in one direction are the stages of the Cognitive Process Dimension, which consists of the six levels of the Taxonomy of Bloom (Remember, Understand, Apply, Analyze, Evaluate and Create) [51]; in the other direction are the stages of the Knowledge Dimension, which consists of the four levels of the Anderson & Krathwohl Taxonomy (Factual, Conceptual, Procedural and Metacognitive) [52]. Figure 6 (based on the graphical model presented in [53]) shows a possible interpretation of this tiered yard where it can be seen that joint progress in both dimensions means not only a cognitive effort (which means moving from moment 1 -in which the student masters the application of a concept- to moment 2 -in which the student is capable of critically analyzing that concept-) but also intellectual commitment (which means going from moment 2 -in which the student is able to critically analyze a concept- to moment 3 -in which the student can take a creative advantage of the previously acquired knowledge to analyze the procedure that brings together different concepts-). to be able to climb each terrace and reach the upper levels in both dimensions. The creativity approach in criticality implies the design of experiences that allow students to be able to climb each terrace and reach higher levels, advancing in both dimensions (with cognitive effort and intellectual commitment).

This model is opposed to the traditional approach of advancing only in the first three vectors of the dimension of knowledge (Factual-Conceptual-Procedural) towards the highest stages of Bloom's Taxonomy, which is known as domain specificity of subjects [54].

We consider that the two-dimensional approach to development is an adaptation of the learning process to the personality characteristics of Gen Z students. This adaptation should be based on recognized theories of cognitive (critical thinking) and metacognitive (creative thinking) processes. Therefore, for this study, we proposed the following research question (RQ):

RQ1: What are the learning experiences that develop in Gen Z students the ability to advance each vector in the stepped playground of their learning?

RQ2: What are the most appropriate learning techniques for Gen Z students to develop skills of both creativity and criticality in the subjects of the engineering program?

## Methodology

### Participants and procedure

In addition to considering the possible bias that the cognitive style introduces in the evaluation of creative thinking, the significant influence of motivational processes should also be considered. On the one hand, the behavior of the groups is strongly modified by the situations of stress and health of the students, as well as by the environment generated by the teacher, the attitudes between peers, the conditions in which the tests and surveys are conducted, and the observations and rubrics that are used. Hence, the importance of conducting experiments with a methodological design appropriate to the social sciences. In the case of the present study, 4-group Solomon research design was chosen [55, 56]. A total of 323 undergraduate students of engineering programs have voluntarily participated in our study: 173 students attended three “creativity in criticality” experiences each semester during three consecutive semesters, so that each student participated in a total of 9 experiences (Experimental group with and without Pre-Test), and 150 students remained untrained (Control group with and without Pre-Test). It is also very important that, in the experimental groups, regardless of whether they have Pre-Test or not, warm-up activities are carried out, designed as in the infusion-immersion mixed approach methodology, to increase the interventions and interactions of the divergent stage of the creative process. Participants who contributed to this study had an average age of 22 years at the time of the PostTest application, and were considered to belong to generation Z (born after January 1, 1995) [3]. The group criteria were:

Total Experimental Group: 173 students.

Experimental Group with PreTest and Treatment: 101 students.

Experimental Group without PreTest, only Treatment: 72 students.

Total Control Group: 150 students.

Control Group with PreTest: 72 students.

Control Group without PreTest: 78 students.

### Instrumentation

**Fig. 7:**
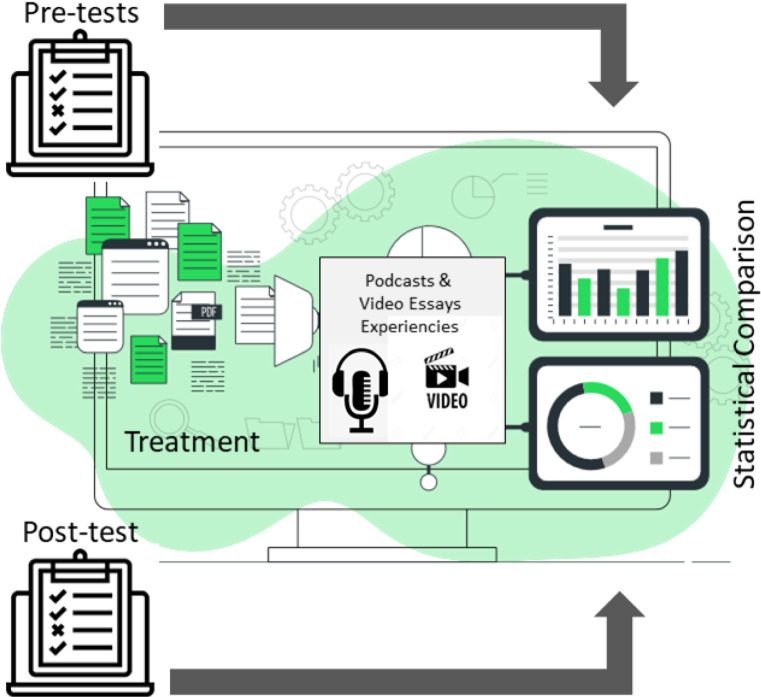
Methodological design

*Instruments for data collection in Pre-Test and Post-Test:* Vocabulary tests, with different levels of complexity, some about general culture and others with specific lexicons of each subject. The lists were made from lists of the SAT Vocabulary [57] and the Corpus of Contemporary American English (COCA) [58]; Reading comprehension tests, designed to determine the level of cognitive maturity of students; modified version of the tests and rubrics presented by Paul & Elder [59][60]; and the AAC&U VALUE Rubrics, developed for the Association of American Colleges and Universities Essential Learning Outcomes [61]. Some definitions of the performance levels of the Capstone, Milestones, and Benchmark rubrics, adapted from the AAC&U VALUE Rubrics, are shown in Appendix A. The pretests were very important to know the level of cognitive maturity of the students. Therefore, Egan's taxonomy was used in this study to place students: mythical, romantic, philosophical or ironic level. It is important to emphasize that the PreTest did not have a diagnostic function in terms of the level of domain-specific knowledge. The PreTests were also used to find out the level of development that students have in: Creativity and seven other transversal skills and competencies of ABET graduation [62]: Teamwork, Self-knowledge, Transfer, Criticality, Knowledge of Cultural Frameworks, Broad Perspective and Taking Risks. The methodological procedure is shown in the scheme of Fig. 7.

The procedure consisted of carrying out pre-tests on 101 students from the experimental group and 72 students from the control group. These tests were conducted during the first week of classes of each semester. During the semester, the Treatment experiences were carried out with the 173 students of the experimental group. Finally, the subsequent tests were carried out on the 323 students. In the Findings and results section, discussions about the statistical tests performed will be presented: comparison between the results of the Pre-Tests of the experimental and control groups; comparison between Pre-Tests and Post-Tests; and comparison between final grades of the subjects and evaluation of competencies.

*Treatment*: For the selection of the experiences that would be carried out with the students of the experimental group, those didactic activities that were endorsed and validated in the literature review were considered. For the design of experiences using Video Essays, the authors took into account the results and suggestions of the studies by Moser and Hlavacs [63], Anas et al. [64] and Zainal et al. [21]**.** For the design of experiences using Podcast, the authors considered the results and suggestions of the studies by Ketelle [65] and Hess et al. [66]. For the design of experiences using World Café, the authors considered the results and suggestions of Torres and Costa Neto [67] and Bisello et al. [68]**.** Various other activities **(**on-line debates, discussions, argumentative panels, round tables and micro-workshops) were carried out to adapt to the particular social conditions of each group and to the circumstances derived from confinement by COVID-19 throughout the year 2020 and part of 2021 [69, 70]. These activities provided opportunities for students to share their opinions and practice collaborative learning activities on the subject, with a student-centered approach. Scaffolding ZPD was incorporated with learning activities designed with the tiered yard.

## Results

### Experimental design

All the "Creativity in Criticality" experiences included in this project were designed so that Gen Z students could holistically and comprehensively develop their creative thinking and critical thinking skills, efficiently taking advantage of their personality traits and characteristics of learning, which were discussed in the previous sections. At all stages of the treatment, their digital information management skills were stimulated, and they were encouraged not to passively wait for an explanation of the experience, but rather to actively participate in training their ability to understand, translate and encode symbolic information and to develop their own concepts and constructions through abstract reasoning. Some of the considerations that were discussed in detail with the instructors were related to the fact that the activities should be carried out with total concentration with the conscious objective of generating an active and collaborative learning environment, where the students were able to get involved in the learning process, being aware of the different levels of cognitive and motivational effort required in each session (according to what is explained in Fig. 6. The students were promptly informed that the process was going to involve increasing efforts, in the sense that the activities they would gradually become more difficult. The students received weekly feedback to know their progress and collaborate in fulfilling that the easier activities would be mastered through repetition before practicing the more difficult ones (scaffolding and ZPD concepts).

### Examples of experiences and activities

The activities were designed considering the actual cognitive stages of thinking of the students according to Kieran Egan’s theory [71, 72], and incorporated analysis sessions. Specific metacognitive tools were considered to develop creative thinking and to enhance the capacity for analysis and combination of existing ideas and images through new disruptive and alternative solutions. Table 1 shows the different activities included in Treatment and the skills to develop in the processes.

**Table 1:** Type of experiences included in Treatment

Type of Experience	Description	Skill to develop
Video Essays and Podcasts	Creation of the scripts by the students (review and feedback by the teacher) and subsequent recording, production, and post-production	Creativity in Criticality
Challenge-based learning	Collaborative experience with experts in industry facilities, energy utilities facilities and technology companies	Taking Risks*
Supervised Questioning Session	The formulation of the questions must be balanced to ensure the inclusion of convergent and divergent questions in the four knowledge domains of the tiered yard	Embracing Contradictions*
Dialogue Seminars	Meeting between a small group of students and a mentor during which students read their essays and the whole group shares their experiences	Attentiveness towards different situations*
Argumentative Panels	Argumentative discourse process in science as part of a repertoire of strategies to support the acquisition of scientific literacy	Self-awareness*
World Café	Academically rigorous data collection method, with an atmosphere of trust and open discussions among participants	Broad Perspective*

*See Appendix A

**Fig. 8:**
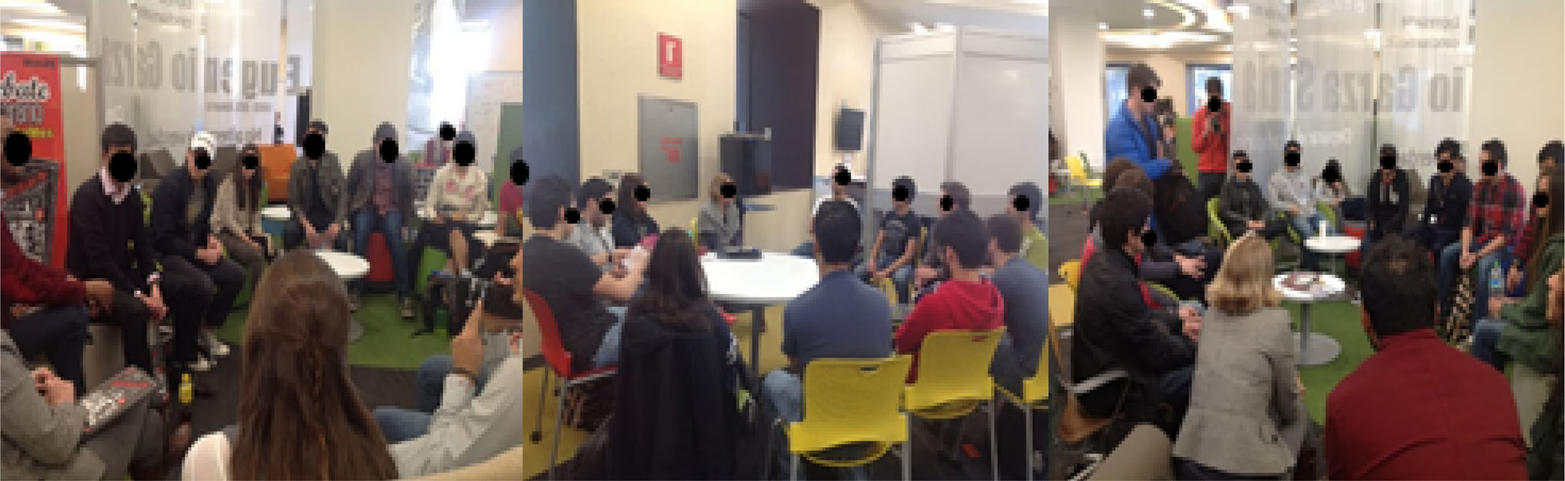
Dialogue seminars

*Dialogue Seminars* [73]. The Dialogue Seminar was a safe space that allowed students to discuss ideas openly. The instructor provided a case example of a real conflict and the group discussed to reach a consensus, sharing opinions and different perspectives, and fostering the students' intellectual engagement with their own learning process. Figure 8 shows the photographs taken during different dialogue seminars with students, where metacognitive instruction strategies were used, and the concepts: Scaffolding and ZPD were used.

*Supervised Questioning Sessions* [74]*.* The supervised question method was used to stimulate the recall of the knowledge acquired in previous sessions and improve the understanding of the concepts. The instructor planned the sessions sequentially, to get the most motivation from the students and prevent them from responding in a hasty way with a premature closure. When using the Creativity in Criticality approach, the method consisted of sessions of ten questions, three of them non-hierarchical, three questions belonging to the Cognitive Dimension of Bloom's Taxonomy, and four questions belonging to the Knowledge Dimension of Anderson's Taxonomy & Krathwohl, as shown in the design of the tiered yard of the Fig. 6.

**Fig. 9:**
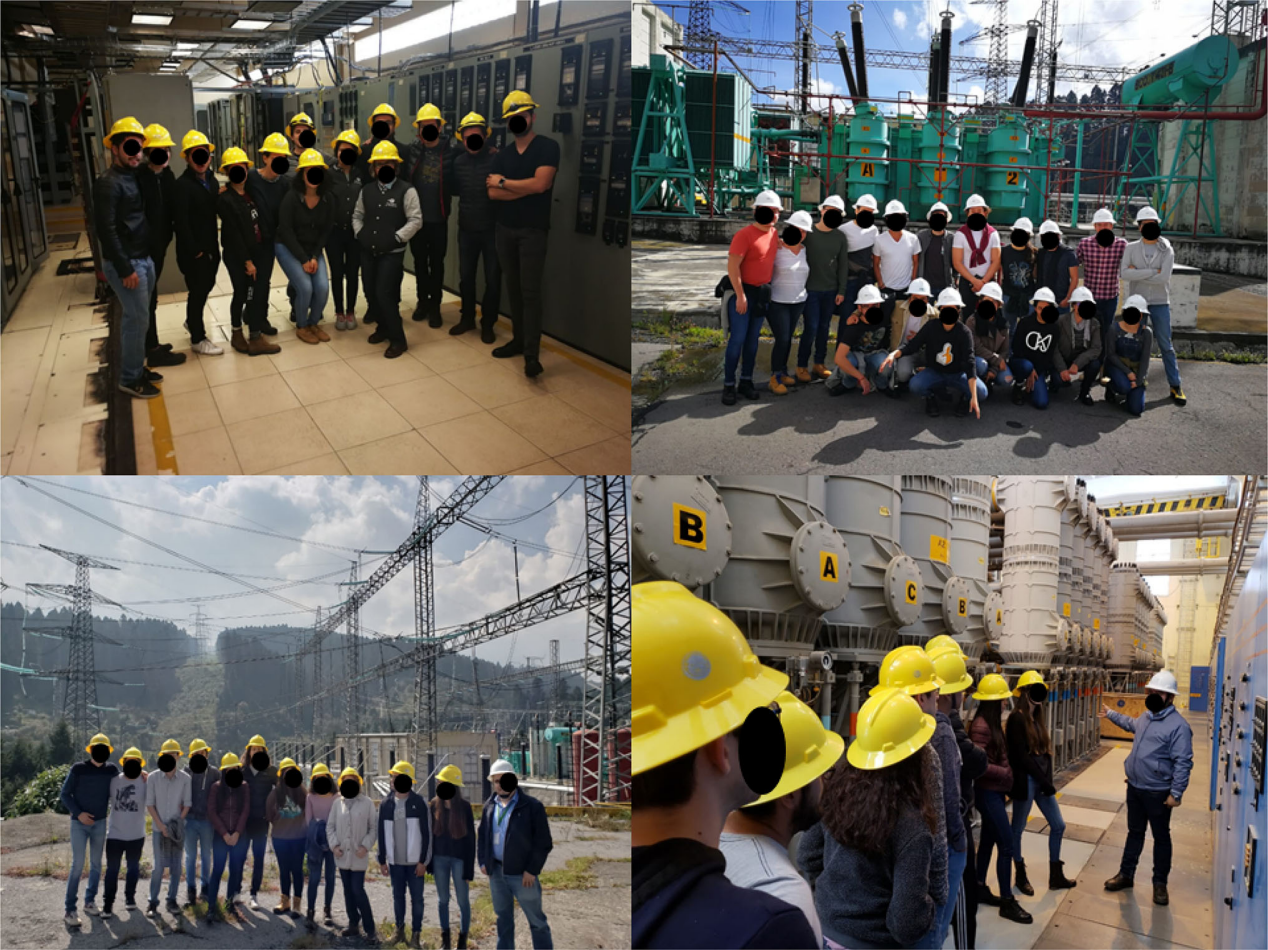
Challenge-based Learning at a 400 kV Power substation

*Challenge-based Experiences* [75]. Challenge-based Experiences (CBL) is an active and experiential learning approach, which allows to develop a real-world perspective "by doing" on a subject of study. This approach offered an ideal learning framework for Gen Z students as it emulates real work experiences in industry and corporations. Figure 9 shows a field trip for a CBL experience in a 400 kV Substation.

### Podcasts and video essays recording (including script writing)

**Fig. 10:**
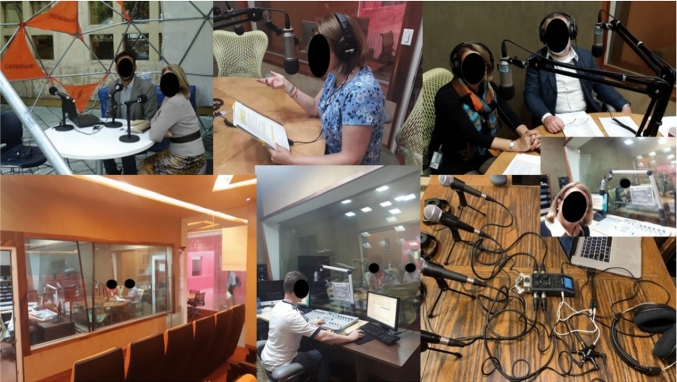
Podcast recording at the radio station and portable recording equipment

Students of the Experimental Group made second-generation Podcasts (Podcast with video) and Video Essays using both screencasts and audio recordings made in the campus radio station facilities and audio recordings made with the portable equipment. The topics developed with this technique included teach other classmates how to use specialized software, record their learnings on a particular topic that shows a step-by-step process, so that other classmates can learn the material at their own pace or catch up on missed sessions, and communicate opinions, facts and ideas on the topics seen in class, their applications, advantages, and current news in the sector of interest. Podcasts shows were recorded at the radio station facilities or using portable recording equipment to broadcast from the classroom and outdoors, as shown in Fig. 10.

**Fig. 11:**
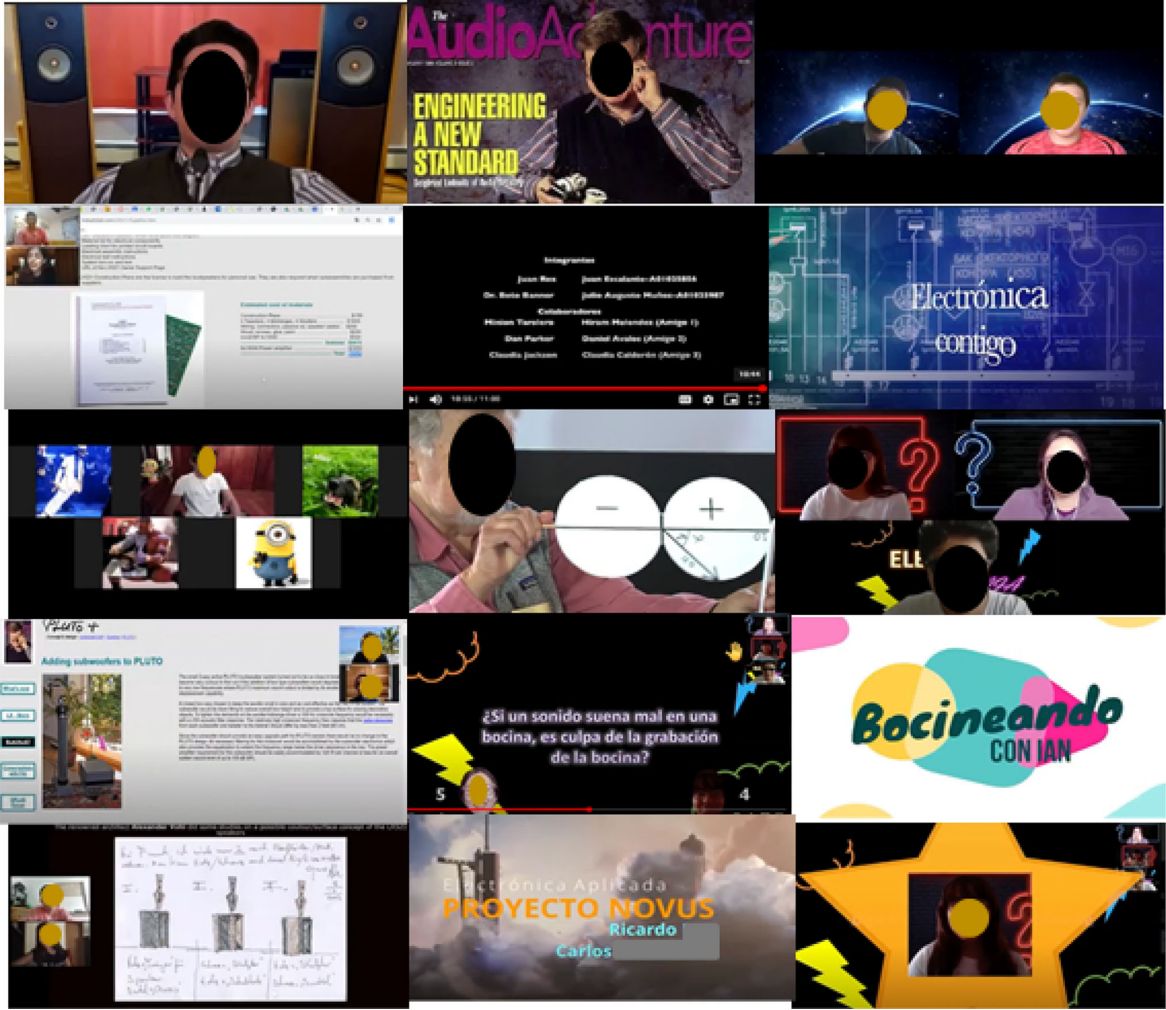
Frame collection of video-essays

The video essay was an attractive tool due to its two main characteristics that make it more appreciated by Gen Z: first, its length, since most video essays do not last more than a few minutes; and second, the video essay is free form because the format and rhetorical strategies can differ enormously from one video essay to another. Another aspect that made video essay attractive to Gen Z engineering students was that there were no specific rules that authors must follow. From the point of view of the teaching–learning process in engineering, the video essays allowed students to be shown things that they could not have been shown in a traditional essay. Furthermore, as the scripts were written following a Serious-Storytelling technique, the images significantly enhanced the story being told: the images were allowed to "speak for themselves". The technological tool chosen for the present study was the recording of video essays and second-generation podcasts (Podcast with video), created from screencasts, with scripts prepared by the students themselves on topics selected from the official syllabus of each course [80]. Figure 11 shows a collection of frames from video essays.

To answer the research question and validate the functionality of the Creativity in Criticality model, the following analyses were carried out:

- Analysis of the results of the Pre-Tests, for 101 students from the experimental group, and 72 students from the control group
- Analysis of the results of the Post-Tests, for the 323 students
- Analysis of the impact of the Treatment on the development of creative thinking and critical thinking for the 173 students of the experimental group
- Discussion on the first RQ: What are the learning experiences that develop in Gen Z students their ability to advance in each vector in the stepped playground of their learning?
- Discussion on the second RQ: What are the most appropriate learning techniques for Generation Z students to develop skills of both creativity and criticality in the subjects of the engineering program?
- Discussion on the results of the opinion survey answered by the students of the experimental group regarding the experiences of the treatment.

### Pre-test analysis

The results of the diagnostic Pre-Test in both groups (101 students from the experimental group, and 72 students from the control group) were compared with the purpose of verifying that the students of both groups had similar initial conditions in terms of the level of development of the following competencies: Taking risks; Embracing Contradictions; Attentiveness towards different situations; self-awareness; and Broad Perspective View. The analysis of the results of the Pre-Tests did not reveal significant differences in their background of skills and competencies.

Regarding the Pre-Tests related to the lexical corpus of the students, the vocabulary tests consisted of proposing to the students the reading of short essays -high-quality lexical texts of less than 200 words- written by specialists. Two different types of tests were implemented to determine, respectively, whether the student could explain the meaning of the words (open response) and whether or not the student could distinguish the meaning of the word in a context. For more details on the design of language and written communication skills tests, see [76].

The study of the results of these previous tests suggests that the sporadic and fragmentary speech habits that Gen Z young people practice in social networks exert an intellectual disintegrative influence on language. Considering these results, we considered that it was particularly important to include, in the Treatment experiences, the exercise and practice of continuity and the ordering of meanings, which is what finally gives coherence to a discourse, whether oral or written.

### Post-test analysis

**Fig. 12:**
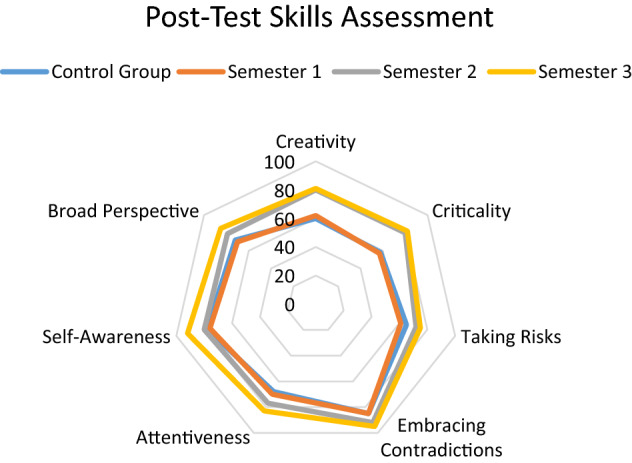
Post-test skills levels measured in semesters 1, 2 and 3

The results of the Post-Test were compared in both groups (173 students from the experimental group and 150 students from the control group) to determine differences in the development of the competencies declared in the Appendix A rubric. Figure 12 shows the performance levels obtained by the control group (average of the 3 semesters of the project) and by the experimental group (in each of the 3 semesters of the project).

The comparison between the average performance of the control group in the three semesters and the performance of the experimental group in the first semester of the project shows similar levels of competence in the skills considered in Appendix A. However, the results obtained by the group experimental in semesters 2 and 3 suggest a significant development in all the skills and an outstanding strengthening in the skills of creativity and criticality. This result is validated with the opinion surveys on the very positive perception of the students of the experimental group towards the Treatment activities (especially those related to the Video essays and the Podcasts).

Post-Test comparison of the skills levels developed in the project revealed that experimental group showed a significantly greater improvement than control group. The Post-Tests using *Association of American Colleges and Universities* (AAC&U) rubrics showed that the experimental group attained 37% improvement in comparison with the students of the control group in the upper “Capstone” level and a 25% decrement in the number of students who remained at the lowest “Benchmark” level of the rubric. In the Milestone 3-level the experimental group attained 65% improvement in comparison with the students of the control group, and in the Milestone 2-level the experimental group attained 34% decrement in comparison with the students of the control group. These results are shown in Table 2.

**Table 2:** AAC&U Rubrics distribution for experimental and control groups

Groups	Value Rubrics			
	Capstone Level	Milestones 2	Benchmark Level	
Experimental Control	22%	33%	25%	20%
	16%	20%	38%	26%
	** + 37%**	+ 65%	− 34%	− **25%**

**Fig. 13:**
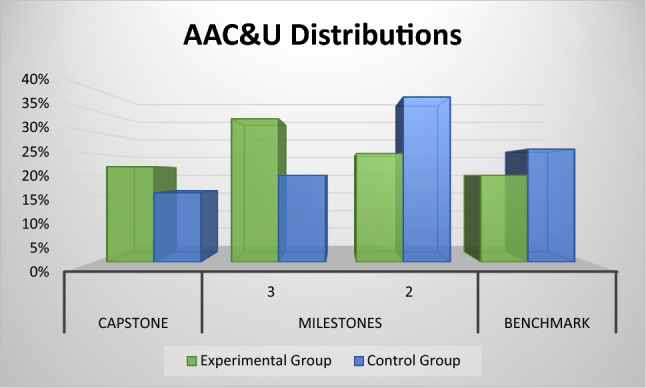
AAC&U distributions of the post-test results

A very interesting analysis of the Post-Test results is that of the behavior of the performance distributions in both groups of students, experimental and control. As shown in Fig. 13, it was observed that the shapes of the Gaussian bells had an asymmetry to the right in the case of the experimental group and a bias to the left in the case of the control group. On the one hand, the left-skewed distribution of the experimental group shows a long tail in the positive direction indicating that most students achieved high performance on the AAC&U rubrics and are in the "Capstone" and "Milestone 3-level" cohorts. On the other hand, the right-skewed distribution of the control group has a long tail to the left, skewed negatively, indicating that most students performed poorly on the AAC&U rubrics and are in the "Milestone 2-level" and "Benchmark" cohorts.

### Treatment impact analysis

**Fig. 14:**
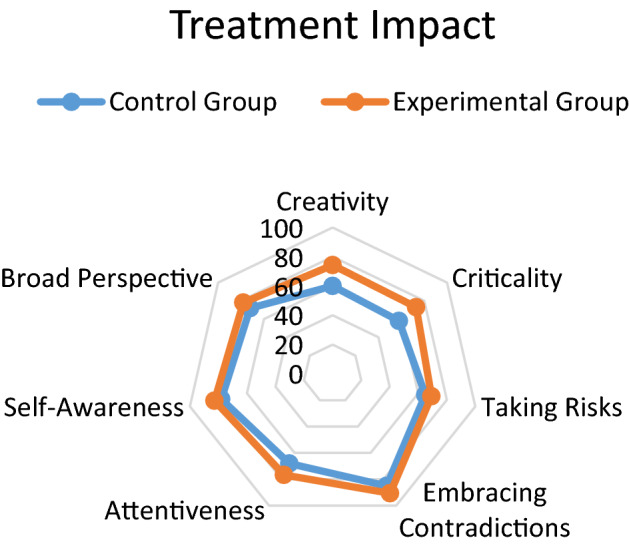
Treatment Impact (in general)

**Fig. 15:**
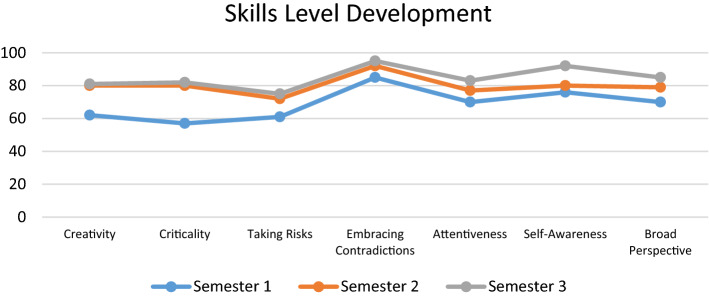
Treatment Impact (in each skills level developed)

With the purpose of evaluating the impact of the different experiences of the Treatment, independent analyzes of each of the competencies investigated were carried out. The treatment impact graph, which can be seen in Fig. 14, suggests high motivation with the use of video essays and podcasts as a means of instruction in the course, in all the competencies evaluated. Regarding the main objective of this work, which was to measure the effectiveness of the use of technological tools for the creation of Video Essays and Podcasts as a treatment for the Creativity in Criticality approach, the following results were obtained:

- Regarding the strengthening of creativity and criticality skills, the results agree with those obtained in the works investigated in the literature review, specifically those of Chaikovska et al*.* [9], Hussein et al*.* [10], Nissenson et al*.*[14], and Becerra & Almendra [13], regarding the advantages of training in communication practices and scientific education with tools of the new digital age through podcast channels. Additionally, the data suggests a high motivation with the use of podcasts as a means of instruction in the course.
- Regarding the efficacy in the use of active and experiential learning techniques related to Challenge-Based Learning, the results showed agreement with the studies by Sgambi et al*.* [7] and Miller et al*.* [8], regarding the relevance of interdisciplinary active experiences for Gen Z students to have greater achievements with the “Taking Risks” skill, enhanced metacognitive awareness and personal motivation, appropriate to their personality traits.
- Regarding the relevance of training with Supervised Questioning Sessions, the results showed a greater understanding of abstract concepts and the development of greater motivation to make cognitive efforts. These results agreed with those obtained by Wu et al*.* [12] and Qamar et al*.* [17] related to the strengthening of students in their development of the competence of "Embracing Contradictions" to overcome cognitive biases related to *Cognitive Fixation* and *Premature Closure*.
- Regarding the development of competencies throughout the three semesters of the project, Fig. 15 shows that not all competencies had the same level of strengthening. The competencies that were strengthened the most were, in the first place, Criticality and, in second place, Creativity. These results agreed with those reported by Torres-Gómez et al*.* [15] and Zúñiga-Robles & Truyol [77], since the improvements in the effectiveness of the approach were made possible by the instructional strategy, especially the discussions and interactive sessions, which were designed to include aspects of critical thinking and creative thinking, as explained in the previous sections.

### Discussion on the first RQ

Regarding which were the learning experiences that best developed in Gen Z students their ability to advance in each vector of the "Creativity in Criticality" taxonomy, the results suggest that the experiences of creating Video Essays and Podcasts (especially those that include video) achieved not only a greater strengthening of skills but also a very good acceptance by Gen Z students. Approximately 76% of students reported perceiving less cognitive effort when acquiring higher-order skills (equivalent to step 1–2 in the tiered yard of Fig. 6) in semesters 2 and 3. Almost 72% of the students reported perceiving greater commitment and motivation when acquiring metacognitive skills (equivalent to step 2–3 in the tiered yard of Fig. 6). Figure 6) in semesters 2 and 3. These findings suggest the efficacy in the Treatment and greatly exceeds those reported in the studies by Anas et al*.* [64], Persada et al. [22] and Magano et al*.* [24].

### Discussion on the second RQ

Responding to the question of what were the most appropriate learning techniques for Gen Z students to develop skills of both creativity and criticality in the subjects of the engineering program, the results mentioned regarding Fig. 14 suggest that the activities of active, collaborative and experiential learning had a great impact on the learning process of the students of the experimental group, who achieved -in 3 semesters- up to 44% strengthening in the case of criticality and up to 31% strengthening in the case of case of creativity. These results confirm studies published by the authors in 2021 [27, 53] and are consistent with the results obtained in recent studies from 2022, such as those by Hiğde and Aktamış [33] and Giuliano et al*.* [41].

### Students’ opinion survey

The study on the opinions of the students at the end of the project showed a good acceptance towards the type of approach in general (with positive opinions in 77% of those surveyed), and a very good acceptance for three of the activities: Podcasts (81% of the participants); Video Essays (79% of respondents) and World Café (76% of respondents). This last result suggests the perception of benefit, in terms of improving oral and written communication, media literacy and broad perspectives, which students seek as job skills.

Qualitative opinion survey, interviews and focus groups that were carried out to interpret students' feelings of online dialogue seminars during the COVID-19 lockdown showed that learning during the pandemic raised many concerns about future adaptations of online learning, especially related to the widespread disappointment of students in their learning effectiveness experiences when the seminars were conducted online. The results were consistent with those obtained in other studies conducted by Park in 2020 [69] and Piyatamrong in 2021 [70].

## Conclusions

One of the areas of opportunity in engineering education is the development of job skills that make students more competitive. Here we study the role that two tools, video, and podcasts, can have in the development of competencies following a systematic and quantitative study. The results show that for generation Z they can constitute a very important didactic strategy for the development of graduation competencies. The significant increase in communication skills, linguistics, as well as cognitive and emotional empathy was evident. The results show that the inclusion of these reflection spaces within the rigid scientific space of the engineering classroom allowed students to arouse their curiosity and the formation of an opinion. The main conclusion that can be drawn is that the Approach to Creativity and critical thinking (Criticality) is an effective cognitive tool to acquire creative thinking and foster the specific temperament dispositions required by Generation Z engineering students. More studies are necessary to study the role of these tools in other engineering environments, however, this study establishes a solid direction to help develop skills using podcasts and videos.

## Appendix A

Example of the Rubric used as PostTest to evaluate skill development. (*This rubric was created using the Association of American Colleges and Universities (AAC&U) VALUE Rubrics)* [78]*.*

**Table:**

Skill to develop	Capstone	Milestones		Benchmark
	4	3	2	1
Taking risks	Going beyond original parameters of assignment, introducing new materials and forms, tackling controversial topics, advocating unpopular ideas or solutions	Incorporates new directions or approaches to the assignment in the final product	Considers new directions or approaches without going beyond the guidelines of the assignment	Stays strictly within the guidelines of the assignment
Embracing Contradictions	Integrates alternate, divergent, or contradictory perspectives or ideas fully	Incorporates alternate, divergent, or contradictory perspectives or ideas in an exploratory way	Includes (recognizes the value of) alternate, divergent or contradictory perspectives or ideas in a small way	Acknowledges (mentions in passing) alternate, divergent, or contradictory perspectives or ideas
Attentiveness towards different situations	Interprets intercultural experience from the perspectives of self and more than one worldview and demonstrates the ability to act in a supportive manner that recognizes the feelings of another cultural group	Recognizes intellectual and emotional dimensions of more than one worldview and sometimes uses more than one worldview in interactions	Identifies components of other cultural perspectives but responds in all situations with their own worldview	Views the experience of others but does so through their own cultural worldview
Self-awareness	Effectively addresses significant issues in the natural and human world based on articulating one’s identity in a global context	Evaluates the global impact of one’s own and others’ specific local actions on the natural and human world	Analyzes ways that human actions influence the natural and human world	Identifies some connections between an individual’s personal decision-making and certain local and global issues
Broad Perspective View	Evaluates and applies diverse perspectives to complex subjects within natural and human systems in the face of multiple and even conflicting cultural positions	Synthesizes other perspectives (such as cultural, disciplinary, and ethical) when investigating subjects within natural and human systems	Identifies and explains multiple perspectives (such as cultural, disciplinary, and ethical) when exploring subjects within natural and human systems	Identifies multiple perspectives while maintaining a value preference for their own cultural positioning
